# Exercise and exercise training‐induced increase in autophagy markers in human skeletal muscle

**DOI:** 10.14814/phy2.13651

**Published:** 2018-04-06

**Authors:** Nina Brandt, Thomas P. Gunnarsson, Jens Bangsbo, Henriette Pilegaard

**Affiliations:** ^1^ Section for Cell Biology and Physiology Department of Biology University of Copenhagen Copenhagen Denmark; ^2^ Section of Integrated Physiology Department of Nutrition, Exercise and Sports University of Copenhagen Copenhagen Denmark

**Keywords:** Autophagy, High‐intensity exercise training, skeletal muscle

## Abstract

Moderately trained male subjects (mean age 25 years; range 19–33 years) completed an 8‐week exercise training intervention consisting of continuous moderate cycling at 157 ± 20 W for 60 min (MOD;* n *= 6) or continuous moderate cycling (157 ± 20 W) interspersed by 30‐sec sprints (473 ± 79 W) every 10 min (SPRINT;* n *= 6) 3 days per week. Sprints were followed by 3:24 min at 102 ± 17 W to match the total work between protocols. A muscle biopsy was obtained before, immediately and 2 h after the first training session as well as at rest after the training session. In both MOD and SPRINT, skeletal muscle AMPK^T^ ^hr172^ and ULK^S^ ^er317^ phosphorylation was elevated immediately after exercise, whereas mTOR^S^ ^er2448^ and ULK^S^ ^er757^ phosphorylation was unchanged. Two hours after exercise LC3I, LC3II and BNIP3 protein content was overall higher than before exercise with no change in p62 protein. In MOD, Beclin1 protein content was higher immediately and 2 h after exercise than before exercise, while there were no differences within SPRINT. Oxphos complex I, LC3I, BNIP3 and Parkin protein content was higher after the training intervention than before in both groups, while there was no difference in LC3II and p62 protein. Beclin1 protein content was higher after the exercise training intervention only in MOD. Together this suggests that exercise increases markers of autophagy in human skeletal muscle within the first 2 h of recovery and 8 weeks of exercise training increases the capacity for autophagy and mitophagy regulation. Hence, the present findings provide evidence that exercise and exercise training regulate autophagy in human skeletal muscle and that this in general was unaffected by interspersed sprint bouts.

## Introduction

Endurance exercise training increases the oxidative capacity of skeletal muscle in part through increased mitochondrial biogenesis in skeletal muscle with concomitant health beneficial effects and enhanced exercise performance (Booth et al. 2012). Thus, exercise training has been shown to increase the content and/or activity of oxidative proteins such as OXPHOS complexes, cytochrome (Cyt) c, and citrate synthase (CS) in human and rodent skeletal muscle (Hood 2001). Moreover, exercise training has been suggested to regulate mitochondrial quality control mechanisms. This includes enhanced autophagy and mitophagy, which is the selective removal of damaged mitochondria (Drake et al. 2016).

Autophagy is exerted by a complex machinery of multiple components. The autophagosome formation is an important step in autophagy and the binding of UNC51‐like kinase (ULK)1 protein and the Class III PI 3‐kinase (PI3K) complex, including Beclin1, has been shown to be involved in formation of autophagosomes (Nakahira and Choi 2013). AMP‐activated protein kinase (AMPK) and mammalian target of rapamycin (mTOR) have been suggested to regulate ULK1 activity, with activation of ULK1 by AMPK through phosphorylation of ULK^Ser317^ and ULK^Ser555^ and inhibition of ULK1 by mTOR through phosphorylation of ULK^Ser757^ (Egan et al. 2011; Kim et al. 2011). One mitophagy model suggests a direct interaction between microtubule‐associated protein 1A/1B‐light chain 3 (LC3) protein on the autophagosome and the mitophagy receptor BCL2 interacting protein (BNIP)3 on the mitochondria (Kubli and Gustafsson 2012). Another model suggests that the E3 ubiquitin ligase protein Parkin recognizes and facilitates ubiquitination of proteins on compromised mitochondria (Kirkin et al. 2009). The autophagy adapter protein p62 links the ubiquitinated mitochondria and the autophagosomes together by interaction with LC3II on the autophagosome membrane, promoting elongation and enwrapping cytosolic cargos including mitochondria (Mizushima and Komatsu 2011). However, the effects of exercise and exercise training on these components in human skeletal muscle are not fully elucidated.

The previous observations that AMPK^Thr172^ and ULK^Ser317^ phosphorylation both increased, while ULK^Ser757^ phosphorylation decreased in human skeletal muscle in response to acute exercise, suggest that exercise enhances autophagy (Schwalm et al. 2015). Similarly, a positive correlation between AMPK^Thr172^ and ULK^Ser555^ phosphorylation has been reported, with increased mTOR phosphorylation and unchanged ULK^Ser757^ phosphorylation in human skeletal muscle in response to 60 min of moderate intensity exercise (Møller et al. 2015). Moreover, Beclin1 and BNIP3 mRNA has been shown to increase in human skeletal muscle in response to acute ultra‐endurance exercise (Jamart et al. 2012). The protein content of p62 has been reported to decrease in both mouse (He et al. 2012; Pagano et al. 2014; Brandt et al. 2017a) and human (Schwalm et al. 2015) skeletal muscle following an acute endurance exercise bout, although others have observed unchanged p62 content after endurance exercise (Jamart et al. 2012; Møller et al. 2015; Fritzen et al. 2016a; Halling et al. 2016). Moreover, previous mouse studies have shown an increase in LC3II protein in response to an acute bout of endurance exercise (Grumati et al. 2011; Jamart et al. 2012; Vainshtein et al. 2015; Halling et al. 2016; Brandt et al. 2017a), whereas the response in human skeletal muscle may be more complex. Hence, a previous study reported a marked increase in LC3b mRNA content in human skeletal muscle in response to acute ultra‐endurance running (Jamart et al. 2012), while other studies with shorter exercise duration observed a decrease in LC3II protein content in human skeletal muscle (Møller et al. 2015; Schwalm et al. 2015; Fritzen et al. 2016a). Furthermore, Masschelein et al. (2014) reported no change in LC3II protein content following acute endurance exercise. Together, this may indicate that the LC3I and LC3II regulation in human skeletal muscle depends on the exercise protocol.

Mouse skeletal muscle Beclin1 protein has been shown to increase with swimming exercise training, while the BNIP3 and Parkin protein content was unchanged (Ju et al. 2016). On the other hand, exercise training in mice has also been reported to increase both BNIP3 and Parkin protein content in skeletal muscle (Brandt et al. 2017b). Furthermore, in LC3II protein increased, while LC3I and p62 protein content was unchanged in mouse skeletal muscle (Brandt et al. 2017a), with exercise training in one study, whereas LC3I and p62 protein increased, and LC3II did not change in mouse skeletal muscle with exercise training in another study (Brandt et al. 2017b). In addition, exercise training responses in p62 and BNIP3 protein in mouse skeletal muscle have been reported to depend on muscle type, because LC3I, LC3II, and BNIP3 increased and p62 protein content decreased in plantaris mucle, whereas LC3I, LC3II, and p62 all increased, and BNIP3 protein was unchanged in soleus muscle with exercise training (Lira et al. 2013).

It is possible that the different observations on autophagy in response to exercise in human skeletal muscle are due to differences in exercise intensity as has been suggested in both humans (Schwalm et al. 2015) and mice (Brandt et al. 2017a). However, the effect of exercise training on the regulation of autophagy in human skeletal muscle as well as the impact of sprint bouts on the regulation of autophagy with acute exercise and exercise training using a matched total work approach remains to be elucidated. Therefore, the aim of this study was to investigate the effect of exercise and exercise training on the regulation of autophagy in human skeletal muscle and examine the impact of sprint bouts on these responses.

## Methods

### Subjects

A total of fourteen healthy, recreationally physically active male subjects volunteered to participate in the study. However, samples from only twelve subjects are used in the present analysis (*n *= 6 in each group), because one subject lacked biopsy material to complete all the analysis and another did not complete the intervention. The average age of the subjects was 25 year (ranging from 19 to 33 year) and the average BMI was 23.9 (ranging from 17.5 to 29.3). The subjects were engaged in 1–3 weekly training sessions (team sports, endurance and/or strength training) but were not involved in regular competition. Participants were fully informed of the experimental procedures of the study and written consent was obtained prior to the study. All study procedures were approved by the Ethics Committee of Copenhagen and Frederiksberg communities and adhere to the principles of the Declaration of Helsinki Title 45, U.S. Code of Federal Regulations, Part 46, Protection of Human Subjects, Revised June 23, 2005, effective June 23, 2005.

### Preliminary testing

Prior to the study all subjects underwent an incremental test to exhaustion on an electronically braked cycle ergometer (Monark 839E, Vansbro, Sweden) to ensure that they had a maximal oxygen uptake (VO_2_‐max) > 45 mL·kg^−1^·min^−1^. The subjects cycled 5‐min at 125 W and 200 W followed by an 25 W·min^−1^ increase in workload until volitional exhaustion defined as pedaling frequency dropping below 50 rpm. In addition, subjects completed a screening, familiarization, and 45 min time‐trial test (TT).

### Experimental protocol

**Figure 1:**
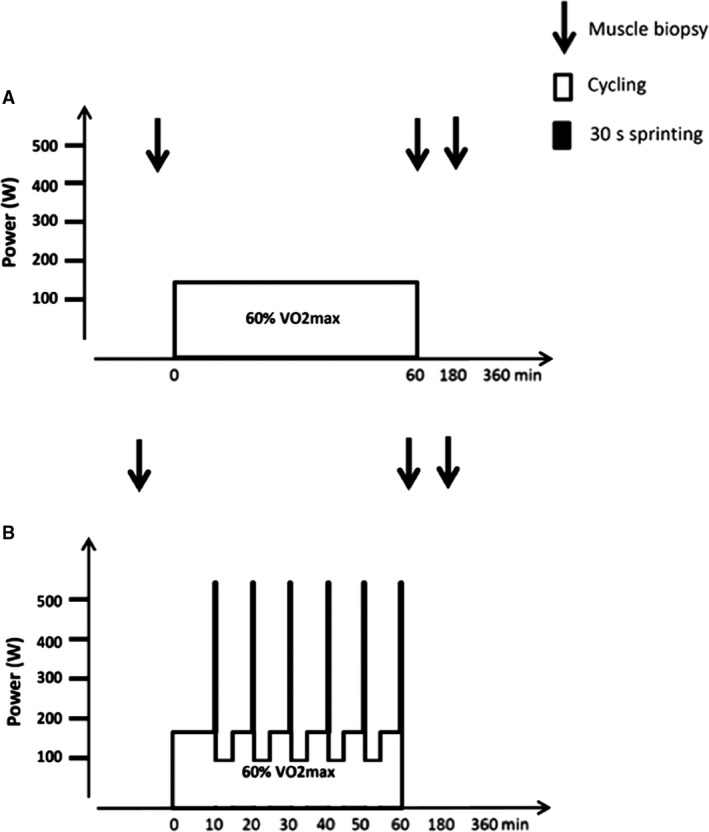
Schematic presentation of the two exercise training protocols consisting of 60 min of cycling (A) continuous cycling with an average power output of 157 ± 20 W eliciting ~60% of VO_2_‐max or (B) continuous cycling for 60 min with an average power output of 157 ± 20 W eliciting ~60% of VO_2_‐max interspersed by six 30‐sec sprints (473 ± 79 W) every 10 min followed by cycling at 102 ± 17 W for 3:24 min. On experimental days, muscle biopsies were taken at rest as well as immediately, and 120 min after exercise.

Based on ranked scores from VO_2_‐max and TT performance, subjects were ranked 1‐14 and were matched in pairs (1st with 2nd, 3rd with 4th etc. until 13th with 14th, and randomly assigned into a continuous moderate intensity training group (MOD; *n *= 6) or a training group with inclusion of sprint intervals (SPRINT; *n *= 6). Subjects completed an 8‐week training intervention of 60 min of cycling 3× per week. During each training session, the MOD exercise training group cycled for 60 min at a constant power output 157 ± 20 W eliciting ~60% of VO_2_‐max. The SPRINT training group performed in each exercise training session cycling for 60 min with an average power output of (157 ± 20 W) eliciting ~60% of VO_2_‐max interspersed by six 30‐sec sprints (473 ± 79 W) every 10 min followed by cycling at 102 ± 17 W for 3:24 min (Fig. 1). Before and after the 8‐week intervention period, subjects completed three experimental days separated by at least 48 h.

During the first and last training session muscle biopsies were obtained. Subjects reported to the laboratory in the morning 2 h after consumption of a self‐chosen standardized breakfast (the same before each trial). During the experimental day, the subjects were allowed to drink water ad libitum but with no food ingestion. A resting biopsy (Pre/Pre Training) was obtained from muscle vastus lateralis followed by completion of the first training session as described for MOD and SPRINT. In addition, a resting muscle biopsy was obtained after 8 weeks of exercise training (Post Training). Muscle biopsies were also obtained immediately (0 h) after exercise and at 2 h of recovery during the first and the last training session. The biopsies obtained after (0 and 2 h) the last training session were obtained for another purpose than the current study and therefore not included in the present analyses. Muscle biopsy samples were immediately frozen in liquid nitrogen and stored at −80°C until further analyses. Blood samples were obtained, but not used in the current study.

### Muscle analyses

A part (~80 mg) of the muscle sample was freeze‐dried for at least 48 h and dissected free of visible blood and connective tissue. Dissection was performed under a stereo microscope with an ambient temperature of ~18°C and a relative humidity below 30%. After dissection, muscle tissue was weighed for lysate preparation.

### Muscle lysates

A part of the muscle sample (~5 mg·d.w.) was used for lysate generation. The muscle tissue was homogenized in ice‐cold buffer (10% glycerol, 20 mmol/L Na‐pyrophosphate, 150 nmol/L NaCL, 50 mmol/L Hepes, 1% NP‐40, 20 mmol/L *β*‐glycerophosphate, 10 mmol/L NaF, 1 mmol/L EDTA, 1 mmol/L EGTA, 20 *μ*g/mL aprotinin, 10 *μ*g/mL leupeptin, 2 mmol/L Na_3_VO_4_, 3 mmol/L benzamidine, pH 7.5) for 2 min at 30 oscilliations per second in a TissueLyser (TissueLyser II, iagen, Valencia, CA, USA). The samples were set to rotate end over end for 1 h at 4°C followed by centrifugation and the lysates were collected as the supernatant. The protein content in the lysate was determined by the bicinchoninic acid method (Pierce Chem, Rockford, IL, USA) and lysates were prepared with sample buffer containing Sodium Dodecyl Sulfate (SDS).

### SDS‐PAGE and western blotting

Phosphorylation levels and protein content were measured by SDS‐PAGE and western blotting using self‐casted gels. PVDF membranes were blocked in 3% fish gel, and protein and phosphorylation sites were determined using primary antibodies against AMPK^Thr172^ phosphorylation (#2535S, Cell Signaling), AMPK*α*2 protein (#G3013, Santa Cruz Biotechnology), Beclin1 protein (#3738, Cell Signaling), BNIP3 protein (#12396, Cell Signaling), LC3A/B protein (#4108, Cell Signaling), mTOR^Ser2448^ phosphorylation (#2971, Cell Signaling), mTOR protein (#2972, Cell Signaling), OXPHOS protein (#110413, Abcam), p62 protein (#5114, Cell Signaling), Parkin protein (#4211S, Cell Signaling), ULK1 protein (#8054, Cell Signaling), ULK^Ser317^ phosphorylation (#12753, Cell Signaling) and ULK^Ser757^ phosphorylation (#6888, Cell Signaling). The membranes were incubated in species‐specific horse radish peroxidase‐conjugated secondary antibodies (Dako, Glostrup, Denmark) and protein and phosphorylation levels were visualized using LuminataTM Classico Western HRP Substrate (Millipore, Denmark). The OXPHOS protein analyses were performed before heating of the samples to prevent that Complex IV was affected. Band intensity was quantified using ImageQuant Las 4000 (GE Healthcare, Munich, Germany) and ImageQuant Imaging software. Protein content and phosphorylation levels were expressed in arbitrary units normalized to control samples loaded on each side of each gel.

### Statistics

Phosphorylation levels and protein content in response to acute exercise and exercise training were evaluated using a two‐way analysis of variance (ANOVA) for repeated measures. If a main effect was observed, a Student‐Newman‐Keul's post hoc test was used to locate differences between time points and training protocols. A significance level of *P *< 0.05 was chosen, and a tendency is reported at 0.05 ≤ *P *< 0.1. Statistical calculations were performed using SigmaPlot Version 12.5.

## Results

### Performance

Exercise training had no effect on whole body VO_2_ max in either group (53.8 ± 5.5 and 54.8 ± 4.8 mL·min^−1^·kg^−1^ before and 53.1 ± 8.3 and 52.3 ± 7.6 mL·min^−1^·kg^−1^ after exercise training in MOD and SPRINT, respectively). However, the work performed during the time trial increased similarly in MOD and SPRINT with exercise training (before: 202 ± 27 and 217 ± 37 J/sec and after: 220 ± 30 and 226 ± 38 J/sec in MOD and SPRINT, respectively).

### Acute exercise

**Figure 2:**
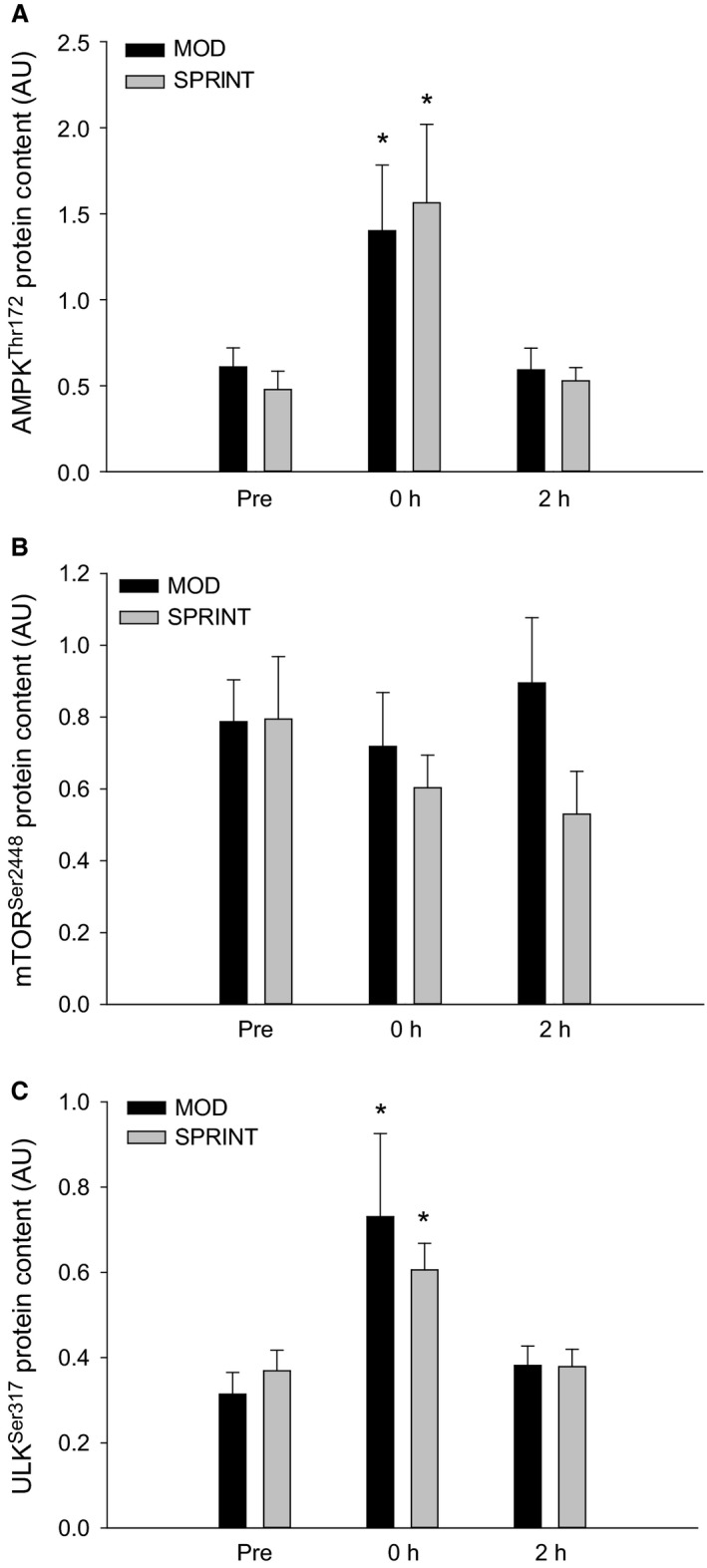
(A) AMPK^T^ ^hr172^_,_ (B) mTOR^S^ ^er2448^ and (C) ULK^S^ ^er317^ phosphorylation Pre, immediately after (0 h) and 2 h after 1 h of continuous moderate intensity exercise at 60% of VO_2_‐max (MOD) or continuous moderate intensity exercise interspersed with sprints (SPRINT). Values are presented as means ± SE;* n *= 6. *Significantly different (*P *< 0.05) from Pre.

In both MOD and SPRINT, skeletal muscle AMPK^Thr172^ phosphorylation was ~2–3 fold higher (*P *< 0.05) immediately (0 h) after than before exercise with no difference between protocols (Figs. 2A, 7). There were no differences in AMPK*α*2 protein content at any of the time‐points either in MOD or SPRINT and there was no difference between protocols (Table 1).

**Table 1:** AMPK*α*2, mTOR, ULK1, OXPHOS complex II, III, VI, and V protein content

	MOD				SPRINT			
	Before Training/Pre	0 h	2 h	After Training	Before Training/Pre	0 h	2 h	After Training
AMPK*α*2	1.05 ± 0.09	0.95 ± 0.09	1.16 ± 0.05	0.92 ± 0.09	0.89 ± 0.09	1.03 ± 0.07	0.98 ± 0.07	0.99 ± 0.14
mTOR	0.78 ± 0.14	0.61 ± 0.11	0.75 ± 0.17	0.54 ± 0.14	0.73 ± 0.16	0.64 ± 0.13	0.59 ± 0.05	0.53 ± 0.13
ULK1	0.98 ± 0.28	0.97 ± 0.17	1.01 ± 0.20	1.43 ± 0.36	1.03 ± 0.27	0.82 ± 0.14	0.83 ± 0.09	0.97 ± 0.20
OXPHOS Complex V	1.06 ± 0.1			1 ± 0.1	1.1 ± 0.1			1.2 ± 0.2
OXPHOS Complex III	0.9 ± 0.06			0.8 ± 0.09	0.93 ± 0.06			0.95 ± 0.09
OXPHOS Complex VI	0.69 ± 0.05			0.63 ± 0.08	0.61 ± 0.04			0.72 ± 0.01
OXPHOS Complex II	0.89 ± 0.08			0.69 ± 0.15	1.01 ± 0.15			0.96 ± 0.14

AMPK*α*2, mTOR, ULK1, OXPHOS complex II, III, VI, and V protein content Pre, immediately after (0 h) and 2 h after 1 h of continuous moderate intensity exercise at 60% of VO_2_‐max (MOD) or continuous moderate intensity exercise interspersed with sprints (SPRINT). Values are presented as means ± SE; *n *= 6.

There were no differences in skeletal muscle mTOR^Ser2448^ phosphorylation (Figs. 2B, 7) or in mTOR protein content (Table 1) at any of the time‐points either in MOD or SPRINT and there was no difference between protocols.

In both MOD and SPRINT, skeletal muscle ULK^Ser317^ phosphorylation was ~1.5–2 fold higher (*P *< 0.05) immediately (0 h) after exercise than before with no differences between protocols (Figs. 2C, 7). There were no differences in ULK^Ser757^ phosphorylation or ULK1 protein content at any of the time‐points either in MOD or SPRINT and there was no difference between protocols (Table 1).

**Figure 3:**
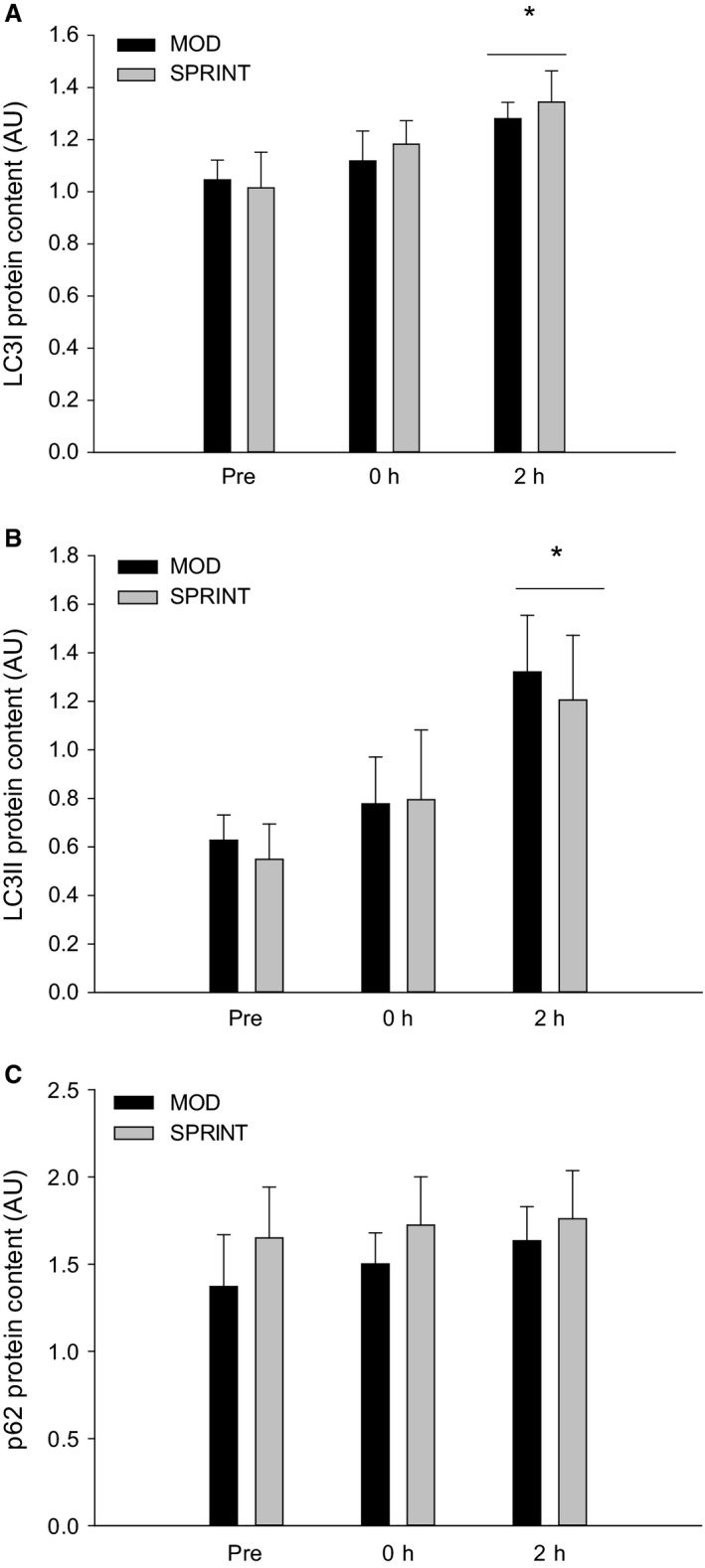
(A) LC3I_,_ (B) LC3II and (C) p62 protein content Pre, immediately after (0 h) and 2 h after 1 h of continuous moderate intensity exercise at 60% of VO_2_‐max (MOD) or continuous moderate intensity exercise at 60% of VO_2_‐max interspersed with sprints (SPRINT). Values are presented as means ± SE;* n *= 6. *Significantly different (*P *< 0.05) from Pre. The horizontal line indicates an overall effect.

LC3I protein content in skeletal muscle was overall ~1.2 fold higher (*P *< 0.05) 2 h after exercise than before and there were no differences between protocols (Figs. 3A, 7).

LC3II protein content in skeletal muscle was overall ~1.5–2 fold higher (*P *< 0.05) 2 h after exercise than before and there were no differences in LC3II protein between protocols (Figs. 3B, 7).

Skeletal muscle p62 protein content was not different at any of the time‐points either in MOD or SPRINT and there were no differences between protocols (Fig. 3C, 7).

**Figure 4:**
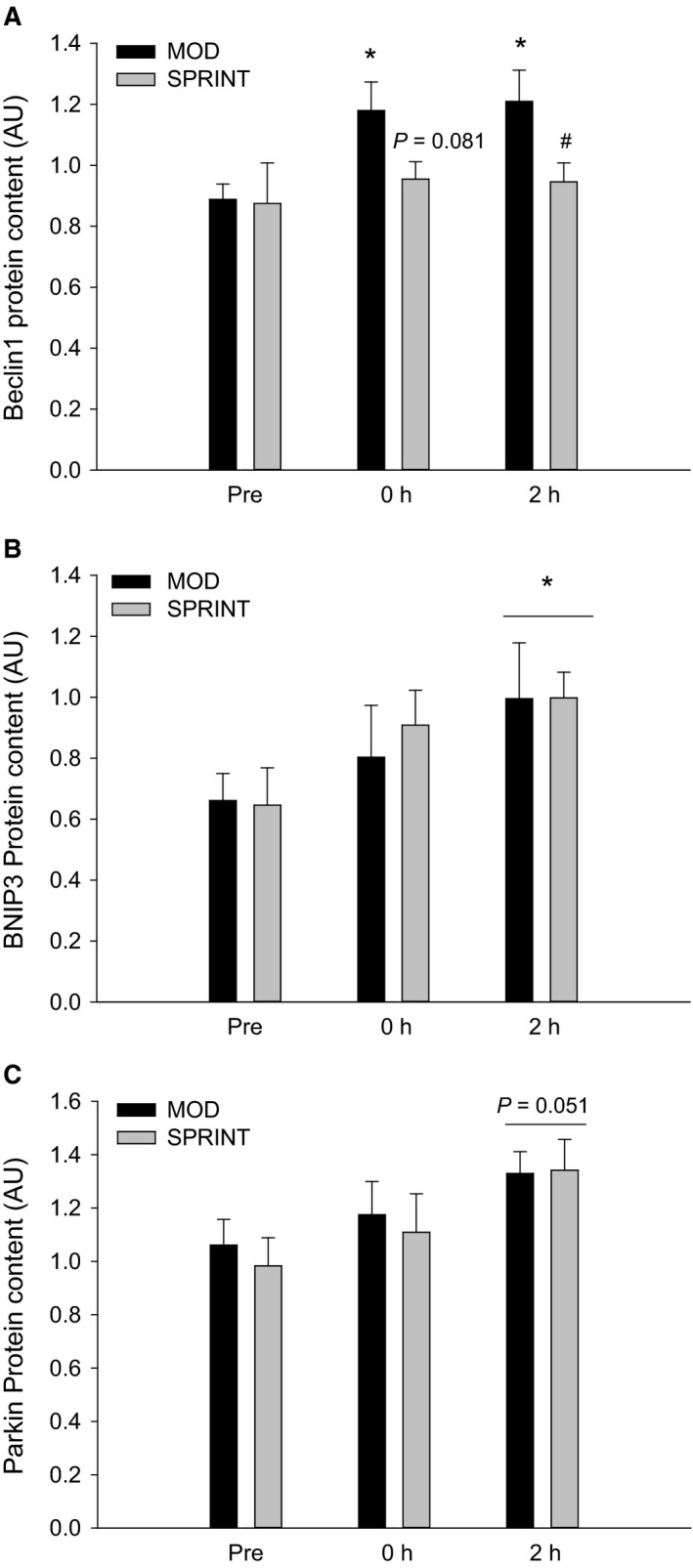
(A) Beclin1_,_ (B) BNIP3 and (C) Parkin protein content Pre, immediately after (0 h) and 2 h after 1 h of continuous moderate intensity exercise at 60% of VO_2_‐max (MOD) or continuous moderate intensity exercise at 60% of VO_2_‐max interspersed with sprints (SPRINT). Values are presented as means ± SE;* n *= 6. *Significantly different (*P *< 0.05) from Pre. ^#^Significantly different (*P *< 0.05) from MOD protocol at given time point. The horizontal line indicates an overall effect. The given *P* value marks a tendency.

Beclin1 protein content in skeletal muscle was ~1.3 fold higher (*P *< 0.05) immediately after (0 h) and 2 h after exercise than before in MOD, while there were no changes in Beclin1 protein content at any of the time‐points in SPRINT. Beclin1 protein content tended to be 25% lower (*P* = 0.081) in SPRINT than in MOD immediately after exercise and was 25% lower (*P *< 0.05) in SPRINT than in MOD 2 h after exercise (Figs. 4A, 7).

BNIP3 protein content in skeletal muscle was overall ~1.3 fold higher (*P *< 0.05) 2 h after exercise than before with no difference in BNIP3 protein between protocols (Figs. 4B, 7).

Parkin protein content in skeletal muscle tended to be higher (*P* = 0.051) 2 h after exercise than before in both MOD and SPRINT and there were no differences in Parkin protein content between protocols (Figs. 4C, 7).

### Exercise training

**Figure 5:**
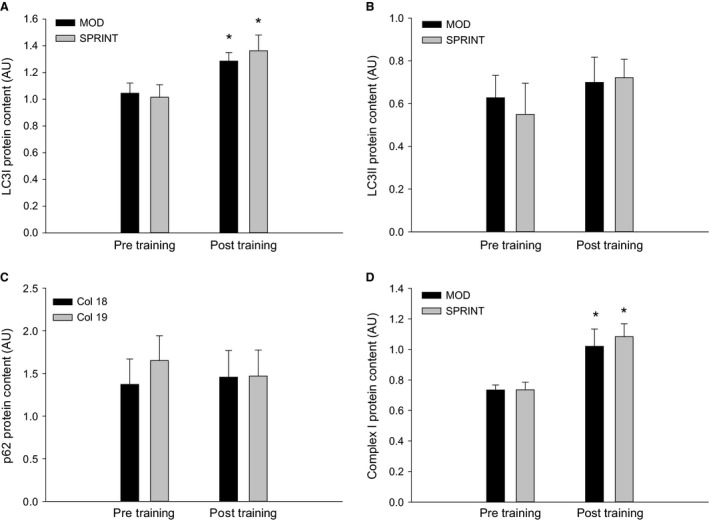
(A) LC3I_,_ (B) LC3II, (C) p62 and (D) Complex I protein content at rest before (Pre Training) and after (Post Training) 8 weeks of exercise training 3 times per week of 1 h continuous moderate intensity exercise at 60% of VO_2_‐max (MOD) or continuous moderate intensity exercise at 60% of VO_2_‐max interspersed with sprints (SPRINT). Values are presented as means ± SE;* n *= 6. *Significantly different (*P *< 0.05) from Pre Training.

LC3I protein content in skeletal muscle was ~1.2 fold higher (*P *< 0.05) after than before the training intervention in both MOD and SPRINT with no difference between protocols (Figs. 5A, 7).

There was no difference in skeletal muscle LC3II protein content before and after the training intervention in either of the protocols and no differences between protocols (Figs. 5B, 7).

There was no difference in skeletal muscle p62 protein content before and after the training intervention in either of the protocols and no differences between protocols (Figs. 5C, 7).

Complex I protein content in skeletal muscle was ~1.2 fold higher (*P *< 0.05) after than before the training intervention in both MOD and SPRINT with no difference between protocols (Figs. 5D, 7). There was no difference in Complex II, III, IV, and V protein content before and after the training intervention in either of the protocols and no differences between protocols (Table 1).

**Figure 6:**
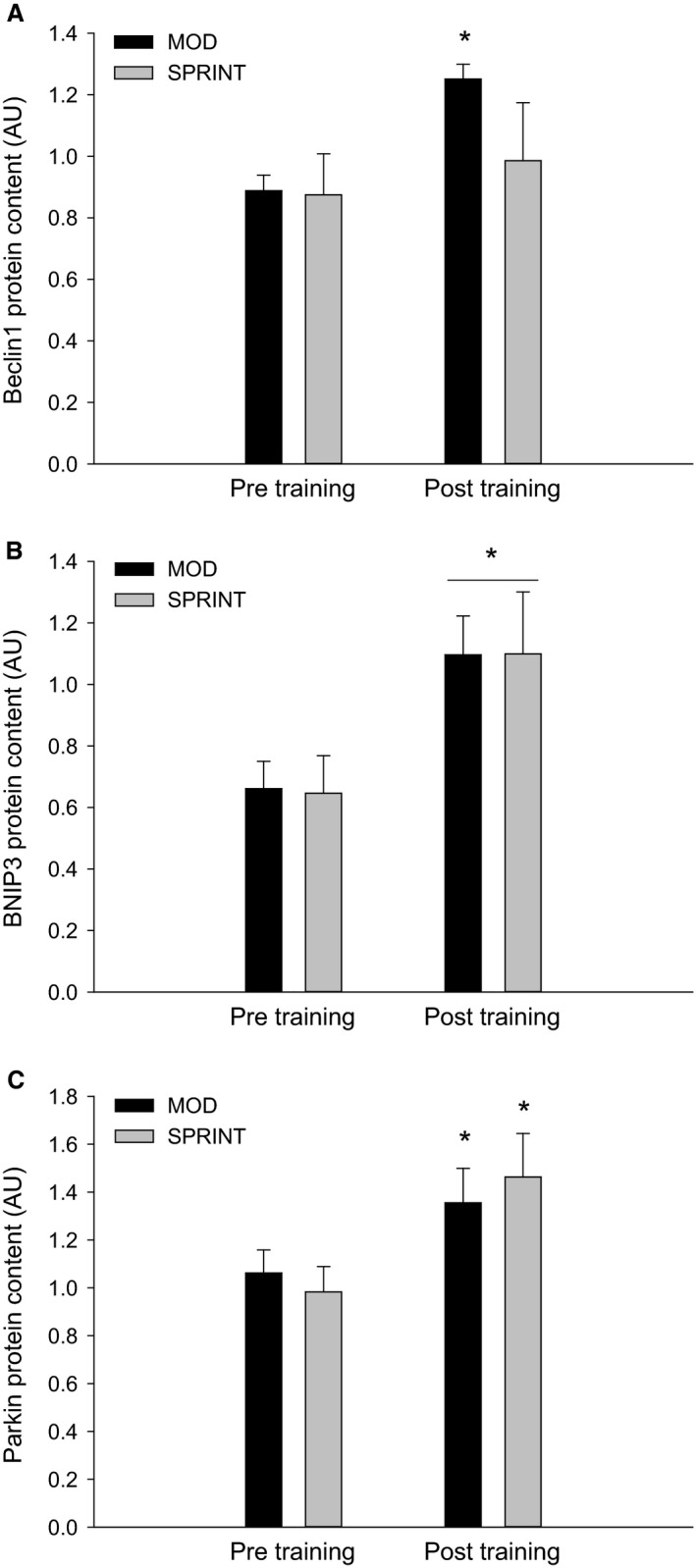
(A) Beclin1_,_ (B) BNIP3 and (C) Parkin protein content at rest before (Pre Training) and after (Post Training) 8 weeks of exercise training 3 times per week of 1 h continuous moderate intensity exercise at 60% of VO_2_‐max (MOD) or continuous moderate intensity exercise at 60% of VO_2_‐max interspersed with sprints (SPRINT). Values are presented as means ± SE;* n *= 6. *Significantly different (*P *< 0.05) from Pre Training. The horizontal line indicates an overall effect.

**Figure 7:**
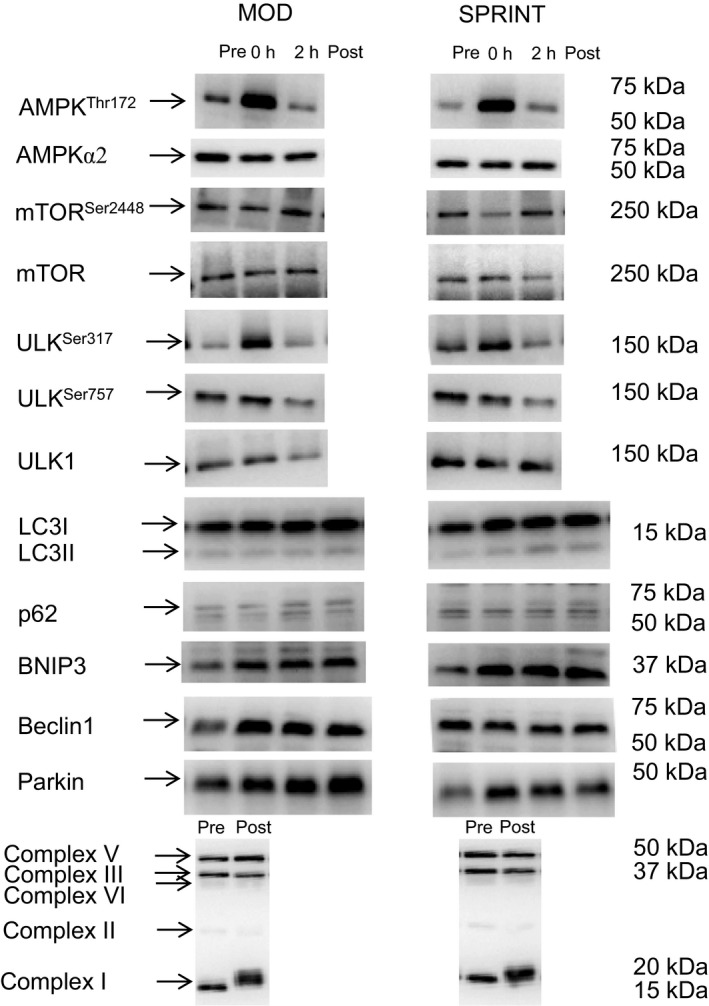
Representive blots at rest (Pre/before training), immediately after (0 h) and 2 h after 1 h of exercise and after (Post) 8 weeks of exercise training 3 times per week of 1 h continuous moderate intensity exercise at 60% of VO_2_‐max (MOD) or continuous moderate intensity exercise at 60% of VO_2_‐max interspersed with sprints (SPRINT).

Beclin1 protein content in skeletal muscle was ~1.3 fold higher (*P *< 0.05) after than before the training intervention in MOD with no difference in SPRINT or between protocols (Figs. 6A, 7).

BNIP3 protein content in skeletal muscle was overall ~1.4 fold higher (*P *< 0.05) after than before the training intervention with no difference between protocols (Figs. 6B, 7).

Parkin protein content in skeletal muscle was ~1.3 fold higher (*P *< 0.05) after than before the training intervention in both MOD and SPRINT with no difference between protocols (Figs. 6C, 7).

## Discussion

The main findings of this study were that a single exercise bout increased LC3I, LC3II, and BNIP3 protein in human skeletal muscle and that 8 weeks of exercise training increased basal LC3I, BNIP3, and Parkin protein content in human skeletal muscle both with continuous moderate intensity exercise and when moderate intensity exercise was interrupted by sprints. On the other hand, including sprint sessions, blunted an increase in skeletal muscle Beclin1 protein content both in response to acute exercise and 8 weeks of exercise training, despite being work matched with the MOD protocol.

### Acute exercise

The current observation that phosphorylation of AMPK and ULK1 at the AMPK‐regulated Ser317 site increased immediately after an acute bout of exercise is in accordance with previous findings (Møller et al. 2015; Schwalm et al. 2015), suggesting increased activation of ULK1 and therefore potential enhanced autophagosome formation. Moreover, the observation that ULK^Ser757^ and mTOR ^Ser2448^ phosphorylation did not change in the present study is in agreement with this possibility and previous studies (Møller et al. 2015; Schwalm et al. 2015). Furthermore, the similar responses in AMPK^Thr172^, ULK1^Ser317^, ULK1^Ser757^ and mTOR ^Ser2448^ phosphorylations in response to continuous moderate intensity exercise and continuous exercise with sprints in the current study are in line with the previously reported intensity independency of ULK phosphorylation (Schwalm et al. 2015). However, the finding that Beclin1 protein content increased in human skeletal muscle in response to the continuous moderately intensity exercise protocol in the present study has not been shown before as no change was reported in a previous study (Møller et al. 2015). This difference between the present and the previous study (Møller et al. 2015) does not seem to be related to the exercise protocol itself using 50% VO_2_ max for 1 h in the previous study and 60% VO_2_ max in the current study. On the other hand, the subjects in the previous study fasted for 36 h prior to the exercise bout, while the subjects in the present study had breakfast the morning at the experiment. This may suggest that substrate availability influences the exercise‐induced regulation of Beclin1 protein in human skeletal muscle. In addition, the finding that the increase in Beclin1 protein was not present in the SPRINT protocol in the present study provides evidence for an intensity‐dependent regulation of Beclin1 protein in human skeletal muscle with sprint bouts blunting the response to exercise. This would be interesting to further explore in future studies. Together this suggests that acute exercise induces autophagy signaling in human skeletal muscle independent of exercise intensity, although factors in the Class III PI 3‐kinase (PI3K) complex involved in autophagosome formation may be inhibited by high‐intensity exercise.

The present finding that acute exercise increased LC3I and LC3II protein in human skeletal muscle is in agreement with previous mouse studies (Grumati et al. 2011; Jamart et al. 2012; Vainshtein et al. 2015; Halling et al. 2016; Brandt et al. 2017a) and in part with a previous study in humans showing an increase in LC3b mRNA content in skeletal muscle in response to acute ultra‐endurance running (Jamart et al. 2012). However, other human studies have observed decreased (Møller et al. 2015; Schwalm et al. 2015; Fritzen et al. 2016a) or unchanged (Masschelein et al. 2014) LC3II protein and no differences in LC3I protein content in skeletal muscle in response to acute exercise. The observed differences between studies in the LC3 response may be related to protocol differences. On the other hand, a study suggested that the autophagy response was intensity‐dependent, because p62 protein content decreased after high‐intensity exercise and not in response to low‐intensity exercise (Schwalm et al. 2015). This observation is not in line with the lack of change in p62 protein content with both exercise protocols in the current study. However, in the previous study the low‐intensity group was cycling for 2 h at 55% of VO_2_ peak, whereas the high intensity group cycled for 2 h at 70% of VO_2_ peak resulting in different total work output (Schwalm et al. 2015), while total work output was matched between the protocols in the present study. Thus, the high‐intensity protocol in the previous study (Schwalm et al. 2015) has demanded a higher energy consumption than the sprint protocol in the present study, which may have contributed to the different response of p62 protein. Furthermore, in agreement with the present study others have reported unchanged p62 content after a single 1 h endurance exercise bout in humans (Møller et al. 2015; Fritzen et al. 2016b). Together this may suggest that acute exercise can increase the LC3 content in autophagosomes and the capacity to exert autophagy regulation independent of exercise intensity and that exercise‐induced autophagy in human skeletal muscle may be more dependent on total work performed than exercise intensity, but further studies are needed to resolve this.

The present finding that acute exercise elevated BNIP3 protein content in human skeletal muscle is novel, but is in agreement with the previous report that BNIP3 mRNA content increased in human skeletal muscle in response to acute ultra‐endurance exercise (Jamart et al. 2012). This suggests that acute exercise upregulates the capacity for mitophagy regulation in human skeletal muscle. Moreover, the similar tendency of Parkin protein to increase at 2 h of recovery in this study further supports that the capacity to regulate mitophagy is affected in recovery from a single exercise bout. Furthermore, the effects of exercise on BNIP3 and Parkin protein followed the same time course as LC3I and LC3II protein indicating a concerted regulation supporting mitophagy in human skeletal muscle in recovery from acute exercise. The observation that the exercise‐induced regulation of BNIP3 and Parkin protein was similar in the two exercise protocols suggests that this regulation is independent of exercise intensity as for LC3I and LC3II protein. Taken together, this suggests that acute exercise increases the capacity for exerting mitophagy and autophagy in human skeletal muscle independent of exercise intensity.

### Exercise training

The present finding that LC3I protein content increased, while LC3II protein content was unchanged with exercise training in both protocols is in accordance with one mouse study (Brandt et al. 2017b), in part with another reporting an increase in both LC3I and II protein (Lira et al. 2013) and different from a mouse study showing an increase only in LC3II protein (Brandt et al. 2017b) and a human study reporting no change (Fritzen et al. 2016a). The observed differences in the LC3 response may be explained by differences in exercise duration and/or intensity. However, the observation that the response was similar in the two protocols in the present study is in line with the exercise intensity independent response reported in mouse skeletal muscle (Brandt et al. 2017a) and suggests that the differences between studies are not due to differences in exercise intensity. Alternatively, the previous observation in mice that LC3II, but not LC3I, protein increased with treadmill exercise training performed twice a day (Brandt et al. 2017a) may indicate that the autophagy response is influenced by the duration of recovery between exercise sessions. Whether this is the case in humans remains to be determined. In addition, the increase in LC3I with exercise training in this study suggests an increased capacity for LC3 lipidation and thereby increased capacity for autophagy regulation.

The present finding that p62 protein content did not change with exercise training in either protocol is in accordance with a previous mouse study (Brandt et al. 2017a), but different from others showing either a decrease (Lira et al. 2013) or an increase (Lira et al. 2013; Brandt et al. 2017b) in p62 protein content with exercise training. It may be suggested that differences between muscle types play a role in these divergent findings, because the p62 protein content decreased in plantaris (mixed fiber type), and increased in soleus (oxidative fibers) in a previous mouse study (Lira et al. 2013). However, p62 protein in quadriceps has both been reported to be unchanged (Brandt et al. 2017a) and to increase (Brandt et al. 2017b) in mouse skeletal muscle with exercise training, suggesting that additional factors also can influence the p62 protein response. Hence, the present observations that LC3I protein increased in both protocols, while LC3II and p62 protein was unchanged with exercise training may indicate that the exercise training did not influence basal autophagy, but enhanced the capacity for LC3 lipidation and thereby the capacity for autophagy regulation independent of intensity. In addition, the increase in Beclin1 protein content in response to exercise training with continuous moderate intensity exercise in the current study is in accordance with the previous observation that Beclin1 protein content increased in mouse muscle in response to swimming exercise training (Ju et al. 2016). However, the lack of effect of exercise training on Beclin1 protein when the moderate exercise intensity was interrupted by sprint exercise provides evidence for an inhibiting effect of short, high‐intensity exercise sessions. This may suggest that bouts of sprint exercise blunt the adaptations in the Class III PI 3‐kinase (PI3K) complex involved in autophagosome formation, but whether this involves other proteins than Beclin1 remains to be determined.

The present findings that BNIP3 and Parkin protein increased in both protocols with exercise training are in accordance with previous findings in mouse skeletal muscle (Lira et al. 2013; Brandt et al. 2017b). Moreover, the previous observation that Parkin protein content in mouse skeletal muscle was unchanged in response to swimming exercise training (Ju et al. 2016) may be due to lower exercise intensity during swimming than during running. On the other hand, the present finding that both continuous moderate intensity exercise and moderate intensity exercise interrupted with sprint bouts elicited a similar increase in Parkin protein in skeletal muscle does not indicate an effect of high‐intensity bouts. Together this suggests that exercise training increases the capacity for mitophagy regulation in human skeletal muscle with similar effects with and without sprint exercise bouts.

The current observations that LC3I, BNIP3 and in part Parkin protein content increased in recovery from acute exercise and increased with exercise training irrespective of protocol are in agreement with the hypothesis that exercise training‐induced protein adaptations can be the result of cumulative effects of transient increases in transcription after each single exercise bout (Neufer and Dohm 1993; Pilegaard et al. 2000). This possibility is further supported by the findings that Beclin1 protein increased with acute exercise and with exercise training only in the protocol without sprints.

The present observations that the exercise training‐induced adaptations in autophagy and mitophagy markers were associated with increases in complex I protein are in line with previous findings in mice reporting an association between adaptations in oxidative and autophagy proteins (Lira et al. 2013). In addition, the observation that exercise performance in the time trial test improved similarly in the two exercise training protocols supports that the reported adaptations in autophagy/mitophagy and oxidative proteins in this study may have had functional importance. Taken together this suggests that exercise training adaptations in autophagy/mitophagy related proteins in human skeletal muscle may play a role in regulating the turnover of an increased content of oxidative proteins, although this remains to be further elucidated.

In conclusion, the present results suggest that a single exercise bout increases autophagosome number, and that exercise training increases the capacity for autophagy and mitophagy regulation in human skeletal muscle. In addition, the present findings provide evidence that these effects are unaffected by interspersed sprint bouts, although regulation of some autophagy markers appears to be inhibited by short lasting high‐intensity bouts.
